# 

**Published:** 1872-11

**Authors:** 


### NOTICES.

"FOOD." William Wood & Co., 27 Great Jones Street, New York, merit the thanks of the public as well as of the profession, by publishing LETHEBY'S "Cantour Lectures on Food," 255 pp. 12mo, treating of the Varieties, Chemical Composition, Nutritive Value, Comparative Digestibility, Physiological Functions and Uses, Preparation, Culinary Treatment, Preservation, and Adulteration of Food.

The mere titular announcement will give the intelligent reader a convincing idea of the great practical value of the book, as it gives the result of accurate scientific investigation, up to the present time.

"Bright's Disease," 148 pp. 12mo., from a homœopathic standpoint, published by Boericke & Tafel, 145 Grand Street, New York; by Dr. JOSEPH BUCHNER of Munich, translated by Samuel Lillienthal, M. D., who, thirty years ago, made his first contribution to homœopathic literature. The pathological view of the malady is of great interest to the physicians of all schools, and merits their attention.

"Journal of Psychological Medicine," treating of the diseases of the nervous system, edited by Prof. HAMMOND of Bellevue Hospital, published quarterly at $5 a year, by D. Appleton & Co., 551 Broadway, New York.

The department of contemporary literature, and that under the heading of "Chronicle," contain a large amount of information of great practical interest to the profession.

One of the very best conducted agricultural weekly newspapers in the United States is the "Farmer's Home Journal," of Lexington, Kentucky, $2 a year.

It contains a large amount of selected home reading, news, science, morals, and domestic teachings, which, for their purity, justness, and wisdom, are well worthy of the commendation of all good men. Nothing silly, or trifling, or vulgar, is ever by any possibility, found in the columns of the "Farmer's Home Journal," while its mechanical execution is as inviting as its moral tone is unexceptionable.

"Hygienic Hand Book," a practical guide for the sick room, by R. T. TRALL, M. D., published by S. R. Wells, 389 Broadway, New York, $2. There is much useful, practical information in this volume, being the result of twenty-five years' practice and observation. The alphabetical arrangement and definitions of subjects add greatly to the convenience of the reader. It is Dr. Trall's best book.

"Medical Electricity." A manual for students, showing the most scientific and rational application to all forms of acute and chronic disease of the different combinations of Electricity, Galvanism, Electro-Magnetism, Magneto-Electricity, and Human Magnetism. By WILLIAM WHITE, M. D., 1 volume, 12mo, pp. 203, muslin; price $2. S. R. Wells, 389 Broadway, New York.

The application of electricity as a remedial agent in cases of disease is a recently developed feature in the progress of medical science, and promises to become one of the most important.

"Scrofulous Affections," with the advantage of homœopathic treatment, described by Dr. H. GOULONS of Weimar, translated by Emil Tietza, M. D., published by Boericke & Tafel, New York and Philadelphia. Educated medical men of all schools can read with profit the general pathology of Scrofulosis, the special forms of Scrofula, etc. The remarks on page 19 demand universal attention, in reference to the perpetuation of scrofulous habits. "The Homœopathic Domestic Medicine," of this same house, is perhaps the most satisfactory for family reference yet issued. 1034 pp. 8vo. Price $5.

Professional readers are directed to the list of medical publications, as the time is approaching for determining what journals to take for the coming year.

MEDICAL PRESCRIPTIONS would not become illegible if a little yellow prussiate of potash were added to common black ink. But if faded, restoration is effected thus: dampen a piece of soft paper, then press it closely on the writing; put a tablespoon of spirits of hartshorn in a tin vessel, with a candle or other blaze under it; hold the soft paper over the fumes of the hartshorn, and the writing will appear on the soft paper; if not plain enough, dampen and repeat the process.

### SOME MEDICAL EXCHANGES.

"American Practitioner," monthly, $3 a year, Louisville, Ky., edited by Professor DAVID W. YANDELL and Dr. THEODORE PARVIN.

"Boston Medical and Surgical Journal," published in monthly and weekly parts for $4 a year, by David Clapp & Son, at 334 Washington Street, Boston, Mass.

"Buffalo Medical and Surgical Journal," monthly, $3 a year, JULIUS F. MINOR, M. D., editor.

"Chicago Medical Examiner," monthly, $3 a year, 797 Wabash Avenue.

"Chicago Medical Journal," monthly, $3 a year, Drs. ALLEN and HAY, editors.

"Dental Cosmos," monthly, $2.50 annually, Philadelphia, Pa., and 767 Broadway, New York.

"Dental Register," monthly, $3 a year, Professor TAFT, editor, 167 Walnut Street, Cincinnati, O.

"Druggists' Circular," monthly, $1.50 a year, Dr. L. V. NEWTON, editor, 36 Beekman Street, New York.

"Eclectic Medical Review," monthly, $2 a year, 166 West 34th Street, New York, Dr. E. S. McLELLAN, editor.

"Good Health," monthly, $2 a year, published by Alexander Moore, 11 Bromfield Street, Boston, and American News Co., 121 Nassau Street, N. Y.

"Gynæcological Journal," devoted to the advancement of the knowledge of the diseases of women; monthly, $2.50 a year, 18 Tremont St., Boston, Mass.

"Homœopathic American Observer," monthly, $2.50 a year, Detroit, Mich.

"North American Journal of Homœopathy," quarterly, $4 a year, G. LILLIENTHAL, editor, 230 West 25th Street, New York, and Trübner & Co., London.

"Herald of Health," $2 a year, issued monthly by Wood & Holbrook, 15 Laight Street, New York. Hydropathic.

"Journal of Applied Chemistry," monthly, $1.50 a year, by Dexter & Co., 17 Spruce Street, New York.

"Journal of Psychological Medicine," being a quarterly review of Diseases of the Nervous System, Medical Jurisprudence, and Anthropology. Edited by WILLIAM A. HAMMOND, M. D., Professor of Diseases of the Mind and Nervous System at Bellevue Hospital; published at $5 a year, by D. Appleton & Co., 551 Broadway, New York, and Trübner & Co., London.

"London Lancet," $5 a year, published monthly, simultaneously with the London edition, by William C. Herald, Esq., agent, 121 Williams Street, New York. Postage 24 cents a year.

"Medical Investigator," monthly, $3 a year, 66 Lake Street, Chicago, Ill.

"Medical and Surgical Reporter" (Dr. BUTLER'S), weekly, $5 a year, 115 South Seventh Street, Philadelphia, and 194 Broadway, New York.

"Medical Times," weekly, $4 a year, published by J. B. Lippincott & Co., 717 Market Street, Philadelphia, Pa.

"Ohio Medical and Surgical Reporter," $1.50 a year, Cleveland, Ohio.

"Phrenological Journal," $3 a year, monthly, published by S. R. Wells, 389 Broadway, New York.

"Science of Health," monthly, $2 a year, by same house.

"Medical Record," semi-monthly, $4 a year, 27 Great John Street, New York.

